# 

**Published:** 1898-08-27

**Authors:** 


					tee hospital. "The standard of highest purity," ?Lancet, August 27th, 1898,
CADBURY'S >> CADBURY'S
cocoa In| cocoa
ABSOLUTELY PURE. NO DRUGS OR ALKALIES USED.
No. 622. Vol. XXIV. [JgSSia Sattjbday,
August 27th, 1898.
[Sab^to.T.tm,,] pEICE TWOPENCE.

				

